# ChatGPT as a Support Tool for Informed Consent and Preoperative Patient Education Prior to Penile Prosthesis Implantation

**DOI:** 10.3390/jcm13247482

**Published:** 2024-12-10

**Authors:** Jacob Schmidt, Isabel Lichy, Thomas Kurz, Robert Peters, Sebastian Hofbauer, Hennig Plage, Jonathan Jeutner, Thorsten Schlomm, Jörg Neymeyer, Bernhard Ralla

**Affiliations:** 1 Charité–Universitätsmedizin Berlin, Corporate Member of Freie Universität Berlin and Humboldt-Universität zu Berlin, Department of Urology, Charitéplatz 1, 10117 Berlin, Germany; till-jacob-valentin.schmidt@charite.de (J.S.); isabel-michaela.lichy@charite.de (I.L.); robert.peters@charite.de (R.P.); sebastian.hofbauer@charite.de (S.H.); henning.plage@charite.de (H.P.); jonathan.jeutner@charite.de (J.J.); thorsten.schlomm@charite.de (T.S.); joerg.neymeyer@charite.de (J.N.); 2 Helios Klinikum Berlin-Buch, Schwanebecker Ch 50, 13125 Berlin, Germany; thomas.kurz@helios-gesundheit.de

**Keywords:** erectile dysfunction, penile prosthesis, ChatGPT, natural language processing, informed consent, patient education

## Abstract

**Background/Objectives**: Artificial intelligence (AI), particularly natural language processing (NLP) models such as ChatGPT, presents novel opportunities for patient education and informed consent. This study evaluated ChatGPT’s use as a support tool for informed consent before penile prosthesis implantation (PPI) in patients with erectile dysfunction (ED) following radical prostatectomy. **Methods**: ChatGPT-4 answered 20 frequently asked questions across four categories: ED and treatment, PPI surgery, complications, and postoperative care. Three senior urologists independently rated information quality using the DISCERN instrument on a Likert scale ranging from 1 (poor quality) to 5 (good quality). Readability was assessed using the Flesch Reading Ease (FRE) and Flesch–Kincaid Grade Level (FKGL) formulas, and inter-rater reliability was measured using intraclass correlation coefficients. **Results**: The inter-rater reliability coefficient was 0.76 (95% CI 0.71–0.80). Mean DISCERN scores indicated moderate quality: 2.79 ± 0.92 for ED and treatment, 2.57 ± 0.98 for surgery, 2.65 ± 0.86 for complications, and 2.74 ± 0.90 for postoperative care. High scores (>4) were achieved for clarity and relevance, while complex issues, such as risks and alternative treatments, scored the lowest (<2). The FRE scores ranged from 9.8 to 28.39, and FKGL scores ranged from 14.04 to 17.41, indicating complex readability suitable for college-level comprehension. **Conclusions**: ChatGPT currently provides variable and often inadequate quality information without sufficient comprehensibility for informed patient decisions, indicating the need for further improvements in quality and readability.

## 1. Introduction

Erectile dysfunction (ED) is a prevalent condition that affects 150 million men globally and is often associated with prostate cancer treatments, such as radical prostatectomy (RP) [1]. ED rates after RP remain high, ranging from 14 to 90%, and often cause emotional distress and a decrease in the quality of life of patients [2]. The etiology of ED in this context is multifactorial, often involving damage to the neurovascular bundles, cavernosal nerve injury and subsequent fibrosis of the penile tissue [2]. Despite advances in nerve-sparing techniques, a significant proportion of patients require medical therapy to restore erectile function. While pharmacological treatments, such as phosphodiesterase-5 inhibitors, offer some improvement, penile prosthesis implantation (PPI) remains the gold standard for those who do not respond to conservative treatments [2]. Prior to PPI, urologic surgeons are often faced with a wide range of questions and concerns regarding the surgery and recovery process. Patients regularly search for information on the Internet early in the process of deciding to undergo surgery before personal consultation with a physician [3]. In the emerging era of artificial intelligence (AI), natural language processing (NLP) models, such as Chat-Generative Pre-trained Transformer (ChatGPT) developed by OpenAI, offer novel opportunities for medical approaches. GPT models are designed for natural language understanding and generation tasks [4]. In addition to improving medical education and automating clinical documentation, potential applications include improving patient–physician communication and providing answers to many of the questions patients have before surgery [5,6]. 

Informed consent is a cornerstone of ethical medical practice, particularly in elective surgeries such as PPI. Patients must fully understand their treatment options, potential risks, and postoperative expectations in order to make informed decisions [5,6]. However, many patients face challenges in comprehending complex medical information, often leading to dissatisfaction or decision regret, despite the best efforts of healthcare providers [4]. NLPs may serve as preparatory or complementary tools to improve patients’ understanding of the procedure and enable them to ask more pertinent questions during the consultation. In this way, AI could serve to deepen patients’ understanding of their therapeutic options and potential risks, enabling a more effective interaction between the surgeon and patient and potentially simplifying the informed consent process. In everyday clinical practice, valuable time can be saved during preparation for surgery. In addition, post consultation, the patient could have any questions that arose later answered at home without having to have another consultation with a surgeon. Moreover, several different use cases for medical approaches have been demonstrated in various studies [7,8,9]. Adams et al. showed that it is possible to automate the conversion of free-text radiology reports into structured templates, and Kienzle et al. identified ChatGPT as an adequate substitute for patient informed consent prior to orthopedic surgery [8,9]. Furthermore, regarding its application in urology, *Xv* et al. revealed that ChatGPT can achieve an accuracy of 94% in predicting the correct diagnosis of common urinary tract diseases from medical reports [10]. Conversely, in a recent evaluation of the quality of information and appropriateness of ChatGPT answers for urology patients, Cocci et al. found a diagnostic appropriateness of only 52%, suggesting that the information provided by ChatGPT to patients must be treated with caution [11]. However, with the increasing availability of NLP models, such as ChatGPT, we may soon experience a paradigm shift in surgeon–patient interactions because of its ability to provide patients with comprehensive information regarding treatment options, complication rates, and perioperative management prior to traditional face-to-face consultations [8]. In the context of the rapidly advancing incorporation of AI into all possible areas of social life, medically validated NLPs could potentially serve as medical counsellors before medical interventions or during hospitalization. During the pre-consultation phase, these tools can educate patients by addressing frequently asked questions about their condition and treatment options, helping them to arrive at consultations that are better prepared and informed [7]. Post consultation, ChatGPT may reinforce and clarify the information provided by surgeons, mitigating the issues of information retention and comprehension often encountered during emotionally charged discussions. This dual role not only enhances patient autonomy and fosters shared decision-making but also supports a more patient-centered approach to care. Simultaneously, surgeons benefit from more efficient consultations, as patients arrive with an informed understanding of their condition and questions. By addressing both patient empowerment and healthcare efficiency, these tools mark a significant evolution in patient–surgeon dynamics [5,6,10]. Although the personal physician–patient relationship is important and will not become obsolete, the implementation of AI models in everyday clinical practice offers a cost- and time-effective opportunity to optimize clinical processes and improve patient experience.

The present study aimed to evaluate the quality and readability of information provided by ChatGPT in response to commonly asked questions by ED patients during preoperative face-to-face consultation prior to PPI, with a focus on the evaluation of the NLP model as a support tool for informed consent.

## 2. Materials and Methods

### 2.1. Collecting Questions

For the present study, a list of questions frequently asked by patients during the preoperative assessment process prior to penile prosthesis implantation (PPI) in our clinical practice, shown in Table 1, was compiled. The questions were derived from frequent inquiries observed during preoperative assessments, with a focus on their clinical relevance and alignment with the typical information needed by patients considering PPI. The questions were not designed as part of a statistically valid data collection process but were based on our everyday clinical experience. Questions were categorized into four thematic areas: treatment options, surgical techniques, complications, and postoperative care. The final set was chosen by the clinical team based on frequency and clinical relevance, with priority given to questions addressing critical aspects of informed consent.

### 2.2. Utilization of ChatGPT

Subsequently, we accessed OpenAI ChatGPT-4.0 via a web browser on March 15th 2024 (state of knowledge until 30 April 2023) and presented all the questions listed in Table 1 to ChatGPT in a single, continuous chat session for each category using basic, nonprofessional English. Prompts were constructed to simulate real-world patient inquiries during preoperative consultations. Each question was input into ChatGPT as a standalone prompt and phrased in natural language, similar to how a patient might express their concerns. To ensure consistency, prompts were standardized to reflect clear and concise language, avoiding ambiguous or overly complex phrasing. For example, the question “What are the risks of penile prosthesis surgery?” was directly input without additional contextual information.

Each query was submitted in the same format, and no follow-up questions were asked to avoid influencing the initial response. This approach allowed for an unbiased evaluation of ChatGPT’s ability to independently generate informative, patient-centered content. A log of all prompts and corresponding outputs was maintained to ensure reproducibility and transparency.

ChatGPT-4.0 was selected for this study as it represented the latest version at the time, with improved natural language understanding, contextual reasoning, and response generation compared to earlier models. These advancements made it the most suitable choice for evaluating AI-driven patient education.

To verify the accuracy and reliability of ChatGPT’s responses, each output was cross-referenced with peer-reviewed articles accessible via PubMed and academic resources available through Google Scholar. Responses were evaluated based on alignment with the established medical literature, and only information consistent with these sources was deemed valid for this study. Priority was given to high-quality, recent publications, including systematic reviews, meta-analyses, and guidelines from reputable medical organizations. In cases where discrepancies arose, the most reliable and up-to-date source was used to determine the accuracy of the response. Ethical approval was not mandatory because no patient was involved.

### 2.3. Standardized Rating Process

The evaluating team consisted of three senior urologic surgeons, each with more than 5–10 years of clinical experience in urology. All surgeons had extensive expertise in preoperative counseling and performing penile prosthesis implantation (PPI), ensuring that their evaluations were grounded in practical, real-world clinical knowledge. Their training included specialization in urological surgery and subspecialty experience in managing erectile dysfunction and conducting patient education during the informed consent process. To ensure objectivity in the evaluation process, an independent coordinator, who was not involved in rating, prepared ChatGPT’s responses. Although ChatGPT is an inherently anonymous AI model, the responses generated in this study were anonymized to remove any identifying details or contextual markers such as timestamps or metadata. This anonymization process was conducted to ensure that independent reviewers evaluating the responses remained unaware of the original context in which they were generated. This approach maintained objectivity and minimized potential bias during the evaluation process. The quality of information provided by ChatGPT was assessed using the DISCERN instrument, a validated and standardized tool with a structured set of 16 questions that assess the accuracy, clarity, reliability, and overall quality of written medical information [8,11,12]. The collected ChatGPT responses were rated independently by each rater, and scores were assigned to each criterion based on the quality of the information provided by ChatGPT, using Microsoft Office Forms^®^ as the survey tool. To ensure unbiased evaluation, the reviewers were not exposed to each other’s ratings. The mean values of the reviewers’ individual ratings were determined for each individual response from ChatGPT. The average of all ratings from the reviewers resulted in the respective mean value of the individual items for each question category.

### 2.4. Readability Assessment

The readability of each output was evaluated using Flesch Reading Ease (FRE) and Flesch–Kincaid Reading Grade Level (FKGL) formulas. FRE scores ranged from 0 (unreadable) to 100 (very easy to read), and FKGL scores corresponded to the US grade level [13]. Microsoft Office Word^®^ was used to calculate the scores for the responses to each question section.

### 2.5. Statistical Analysis

To ensure robustness and coherence in our scoring process, inter-rater reliability was assessed by calculating intraclass correlations. The statistical analysis of the data was performed using Microsoft Office Excel^®^ and IBM SPSS Statistics^®^ 29.

## 3. Results

In the present study, we asked ChatGPT to answer a carefully designed set of questions reflecting those commonly asked by patients in preoperative consultations prior to PPI regarding possible treatment options for ED, the surgical technique of PPI, possible complications, and postoperative care (Table 1). These categories were chosen on the basis of common patient concerns and the need to provide comprehensive information relevant to the informed consent process.

The inclusion of the surgical steps category is noteworthy, as it also plays a critical role in addressing patient concerns, although such details may traditionally be more relevant for medical education. Many patients experience anxiety regarding surgical procedures. By providing a simplified explanation of the steps involved in the surgery tailored to a patient audience, this category helps to demystify the process and set realistic expectations.

**Figure 1:**
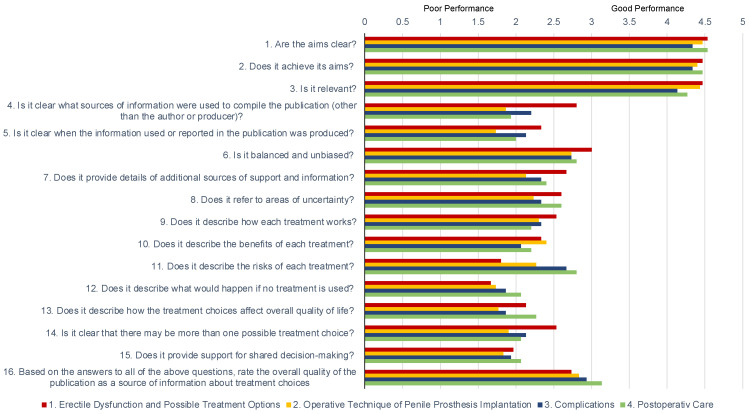
The mean scores for each of the DISCERN items based on ratings from three independent reviewers of ChatGPT’s responses across four categories: 1. erectile dysfunction and treatment options (red), 2. operative technique (yellow), 3. complications (blue), and 4. postoperative care (green) are shown. The scores demonstrate variability in quality among different aspects of the responses, with high consistency in areas such as clarity of aims, while items related to the sources of information or risks of treatment exhibit greater variability and lower scores across sections.

The responses of ChatGPT were rated using a Likert scale from 1 (poor quality) to 5 (good quality) on the DISCERN instrument. The assessment of inter-rater reliability by intraclass correlation showed a coefficient of 0.76 (95% CI 0.71–0.80), indicating solid agreement among our panel of experts regarding the rating of the ChatGPT responses. Detailed results of the mean ratings for each item are presented in Table 2 and Figure 1.

Overall, mean scores across all categories were 2.79 ± 0.92 for responses to ED- and treatment-related questions, 2.57 ± 0.98 for surgery-related questions, 2.65 ± 0.86 for complication-related questions, and 2.74 ± 0.90 for postoperative care-related questions.

For ChatGPT responses to ED- and treatment-related ChatGPT questions, mean scores on Section A (items 1–8) of the DISCERN instrument, assessing the reliability of the provided information, ranged from 2.34 ± 0.98 to 4.53 ± 0.52. For surgery-related questions, mean scores on Section A ranged from 1.73 ± 0.46 to 4.47 ± 0.52. For complication-related questions, mean scores in section A ranged from 1.87 ± 0.74 to 4.34 ± 0.49, and for postoperative care related questions, mean scores ranged from 2.00 ± 0.76 to 4.54 ± 0.52.

The items in Section B (items 9–15) aimed to assess the quality of information provided by ChatGPT. Responses to ED and treatment-related questions revealed mean scores ranging from 1.67 ± 0.52 to 2.54 ± 0.75. Scores for responses to surgery-related questions ranged from 1.73 ± 0.59 to 2.40 ± 0.89 in Section B and responses to complication-related questions had mean scores from 1.87 ± 0.66 to 2.67 ± 1.11. Responses to postoperative care-related questions showed mean scores from 2.07 ± 0.88 to 2.80 ± 1.08.

Section C (item 16) evaluated the reviewers’ perceptions of the overall quality of the responses generated by ChatGPT. The mean score for responses to ED- and treatment-related questions was 2.74 ± 0.75, while the mean score for responses to surgery-related questions was 2.83 ± 0.75. The mean score for responses to questions related to complications was 2.94 ± 0.88, while the mean score for responses to questions regarding postoperative care was 3.14 ± 0.64.

The DISCERN scores demonstrated variability across categories, with ‘Treatment Options’ receiving the highest ratings and ‘Complications’ scoring the lowest. Examples of high- and low-quality responses illustrate ChatGPT’s performance. For “What are the non-surgical treatments for erectile dysfunction?” (Treatment Options), ChatGPT provided a clear, comprehensive answer, mentioning medications, lifestyle changes, and psychological counseling, making it highly suitable for patient education. In contrast, for “What are the risks of penile prosthesis surgery?” (Complications), ChatGPT’s response—“Risks include infection, pain, or device malfunction”—was overly general and lacked depth, omitting key details like infection rates and mitigation strategies. These examples highlight ChatGPT’s strengths in addressing general queries and its limitations with complex topics.

To provide additional context for the DISCERN scores, a qualitative summary of the raters’ rationales for selected criteria has been included in Supplementary Table S3. For example, the moderate score of 3 for “6. Is it balanced and unbiased?” reflects concerns that responses often emphasized treatment benefits while underexplaining risks or alternatives. Similarly, for “5. Does it describe the risks of each treatment?”, raters noted that while common risks were consistently mentioned, severe or rare complications were often omitted, highlighting gaps in the comprehensiveness of ChatGPT’s responses.

The analysis of the FRE and FKGL scores for the responses provided by ChatGPT revealed variations in readability and complexity across the different subsections. The FRE scores ranged from 9.8 for ED treatment-related questions to 28.39 for postoperative care-related questions, indicating an extremely difficult reading level (Table 3). The mean FRE score across all subsections was 18.75 (±7.62). This suggests that the language used in the responses may not be easily comprehensible for a general patient population, particularly those with lower health literacy. These findings align with the Flesch–Kincaid Grade Level (FKGL) results, which also indicated that the responses required advanced reading skills: The FKGL scores, reflecting the educational grade level necessary for comprehension, spanned from 14.04 to 17.41 (Table 3), with an overall mean FKGL score of 15.91 (±1.40).

## 4. Discussion

With the advent of the era of artificial intelligence, and in particular NLP models such as ChatGPT, a plethora of possible new areas of application are emerging in the field of medicine [4,6]. Instead of general Internet searches, patients can also ask for specific information and knowledge about their diseases or planned surgical procedures. Usually, patients are advised about their decision and questions concerning PPI, and the possible risks of the procedure are answered during medical consultation. In the present study, we assessed ChatGPT’s use as a support tool for obtaining informed consent. Therefore, the quality and readability of ChatGPT’s responses to patients’ frequently asked questions in face-to-face medical consultations regarding ED, possible treatment options for ED, surgical techniques of PPI, possible complications, and postoperative care were evaluated.

### 4.1. Quality of Information

The DISCERN instrument, a validated tool for assessing the quality of written health information, showed that the responses generated by ChatGPT varied across different categories of questions. The overall rating undulated around 2.6, and the reviewers’ rating of the quality of information (Section C) hovered around 3 on a scale of 1 (poor quality) to 5 (high quality) across the four categories, indicating the moderate quality of information [12]. This variability was particularly pronounced when comparing responses related to ED and different treatment options (2.74 ± 0.75) with those related to postoperative care (3.14 ± 0.64). ChatGPT’s responses to items 11 and 12 (risks of treatment and risks of no treatment) in the ED and treatment options category received the lowest score of 1.8 ± 0.41 and 1.67 ± 0.52. The highest scores of over four in each category were achieved for points 1–3, which assess the clarity and relevance of the information provided. This suggests that, while ChatGPT can provide accurate and reliable information in certain contexts, it often falls short in terms of quality when more complex or nuanced topics are addressed. The quality of the ChatGPT outputs is inherently tied to the quality of the data that it accesses from Internet-based sources. As noted in prior research, the reliability of Internet-derived health information is often variable, and this inconsistency can propagate into AI responses [14]. This challenge is particularly relevant for NLP models, which rely on publicly available information that may lack the rigor of the peer-reviewed medical literature. Similarly, in the study by Talyshinskii et al. on ChatGPT’s use as a clinical decision maker for urolithiasis and compliance with current European Association of Urology (EAU) guidelines, the model for initial diagnosis and treatment planning is quite consistent with the EAU guidelines, but there are significant gaps in the understanding of surgical planning and metaphylaxis, as the guidelines are not part of the training data [15]. However, possible reasons could also lie in a mismatch between the questions of the DISCERN instrument and those related to the information provided by ChatGPT. For example, if no other treatment options are asked about, the NLP does not provide an answer to these questions and must therefore be classified as insufficient by the reviewer. Nevertheless, in a similarly structured study on ChatGPT’s use as a substitute for informed consent to patients prior to total knee arthroplasty, Kienzle et al. reported higher DISCERN scores of 3.3 to 5 overall and 3.7 to 5 specifically for items 11 and 12 [8]. Furthermore, our results differ from those of Shayegh et al., who rated 70% of ChatGPT responses to patient questions about inflatable PPI as excellent, 20% as satisfactory, and 10% as unsatisfactory, thus requiring substantial clarification [16]. One reason for the differences could be that, in our study, more specific questions were asked about ChatGPT, requiring a more detailed response than that in the study by Shayegh et al. In addition, the divergent evaluation of ChatGPT responses could be due to differences in the reviewers, though it is not clear from the publication by Shayegh et al. how many reviewers participated in the evaluation and what their backgrounds were. Although the rating of the responses of ChatGPT depends on the individual preferences and assumptions of the reviewers and their institutions, the rating in our study was performed by reviewers from two independent institutions, and the intraclass correlation coefficient of 0.76 (95% CI 0.71–0.80) suggests a robust inter-rater reliability. Our results are consistent with those of Whiles et al., who showed that only 54% of ChatGPT responses to patient queries on a variety of urologic topics can be considered good-quality content according to the modified brief DISCERN instrument that they applied [17]. Furthermore, our results are consistent with those of Erkan et al., who showed that ChatGPT has only moderate quality in the DISCERN instrument when answering the most frequently asked questions regarding the treatment of urogenital malignancies [18].

### 4.2. Readability

To avoid misunderstandings, the information provided by ChatGPT should be accessible to non-medical professionals at all levels of education. The FRE ranged from 9.8 to 28.39, indicating a complex readability level, and the FKGL ranged from 14.04 to 17.41, which is likely understandable for college students [13]. Consistent with this, Cocci et al. found FRE scores ranging from 15 to 23 and FKGL scores ranging from 15.6 to 16.1 in their study on the quality of information regarding ChatGPT responses in urology patients [11]. Concordantly, Şahin et al. reported a mean FKGL score of 14.3 ± 1.7 and a mean FRE score of 23.1 ± 7.8 of ChatGPT in their study on the responses of different AI chatbots to the most searched queries about ED [19]. These results indicate that the complexity of the language used by the ChatGPT significantly exceeds the American Medical Association’s recommended readability of medical material for patients, ranging from sixth to eighth grade levels [20]. Accordingly, patients with a lower level of education may not understand the information correctly, even if it is of high quality. However, this is a mandatory requirement for informed consent from patients prior to surgery. In this context, the role of health literacy in patient comprehension is critical for understanding the limitations of tools such as the ChatGPT. Bhalla et al. showed that health literacy significantly impacts patient understanding of PPI [21]. Patients with lower health literacy are more likely to struggle with grasping critical information even when it is presented in simplified formats. This underscores the importance of ensuring that AI-generated content is not only accurate but also accessible to individuals across varying levels of education and literacy.

### 4.3. Implications for Patient–Physician Communication

Our findings suggest that while ChatGPT can be effective in various scenarios in the fields of urology education, research, and practice, as demonstrated in the literature review by Solano et al., its reliability as a source of medical information regarding PPI is still limited [7]. Although NLP models such as ChatGPT may enhance preoperative consultations by providing basic preliminary information, the variability in the quality of more complex information necessitates a cautious approach. It can be concluded that ChatGPT cannot be an exclusive substitute for informed consent prior to PPI, especially when the vast majority of patients have no prior medical training. Personal medical consultation remains absolutely necessary [7,17].

### 4.4. Future Directions for Improvement

To improve the quality of the information provided by NLP models, future efforts should focus on improving the underlying algorithms and training datasets. Incorporating feedback from healthcare professionals and patients may help to refine the models to better meet the needs of patients. In addition, simplifying the language used in the response information can make the content more accessible to medical nonprofessionals. To enhance the utility of ChatGPT as a supportive tool for patient education, future studies could explore the development and validation of standardized prompts designed for healthcare professionals and patients. These prompts could help to streamline interaction and improve response accuracy, ensuring the consistent and effective use of ChatGPT in medical contexts.

### 4.5. Limitations

Our study has several limitations. First, the assessment was performed by only three urologic surgeons, which may not reflect a comprehensive medical point of view, and the questions were not designed as part of a statistically valid data collection process but were based on our everyday clinical experience. Second, the DISCERN instrument was originally developed to evaluate healthcare websites and not NLP-generated information. Future research could be enhanced by developing specifically tailored instruments to assess AI-generated responses. Moreover, the way that patients frame their questions may differ from the format of our study, and comparative evaluations of other LLMs are needed to determine which model is the most effective for providing accurate medical information. One of the limitations of this study was the inherent variability in the quality and readability of ChatGPT responses, which stems from the nature of the tool itself. ChatGPT is designed to generate information based on its training data, and its outputs are influenced by the phrasing of input queries and lack of specific constraints on its responses. This variability was a deliberate focus of our study, as it reflects real-world patient interactions with the tool in its current state. Patients using ChatGPT did not have access to fine-tuning options or quality controls, and our evaluation was intended to simulate these uncontrolled, real-world scenarios. The variability observed in our results highlights both the potential and limitations of using ChatGPT as a support tool for informed consent. Although the tool demonstrates promise in addressing straightforward questions with clear and concise information, its performance declines when handling complex or nuanced topics. Applying constraints, such as predefined prompts or structured outputs, could enhance the consistency and accuracy of ChatGPT responses, thereby influencing surgeons’ evaluations of dimensions such as clarity, balance, and comprehensiveness. While this approach allowed us to evaluate ChatGPT’s performance in its default configuration, the absence of tailored prompts may have contributed to the variability in response quality. More detailed prompts could guide ChatGPT to focus on particular aspects of a question, such as risks, benefits, or mitigation strategies, thereby generating more comprehensive and patient-centered outputs. Furthermore, including instructions such as, “Please explain this in simple terms, as if you were talking to an 8th grader,”, “Use simple language suitable for someone without medical knowledge.”, or “Avoid technical terms unless you explain them first.” could help to align the responses with established readability recommendations for patient-facing materials. 

This underscores the need for future advancements in NLP models, including improved algorithms, curated medical datasets, and mechanisms to enhance the consistency and reliability of the responses. Moreover, future studies should ask patients to assess the readability, comprehension, and overall impact of ChatGPT-based tools for decision-making. In addition to these advancements, future research could further evaluate ChatGPT’s potential as a patient education tool by categorizing DISCERN-based questions into three groups: strengths, correctable weaknesses (addressable through prompt engineering), and inherent weaknesses (not easily mitigated by prompt engineering). This approach would provide clearer insights into the model’s capabilities and limitations. Correctable weaknesses, such as a lack of specificity, could be improved with tailored prompts, while inherent weaknesses, like inaccuracies from training data, may require updates to the model itself. Such a framework would complement this study’s findings and guide targeted strategies for improvement.

## 5. Conclusions

In conclusion, while ChatGPT has demonstrated its potential as a supportive tool in various medical settings, the quality of the information that it provides is variable and often insufficient to support fully informed patient decision-making. This AI model will require additional training before it can be used consistently and reliably as a support tool for informed consent prior to PPI.

## Figures and Tables

**Table 1:** Commonly asked questions in preoperative management prior to penile prosthesis implantation.

Erectile Dysfunction After Prostatectomy and Possible Treatment Options
What are the most common causes of erectile dysfunction after a prostatectomy?
What non-surgical treatment options are available for erectile dysfunction after a prostatectomy?
How effective are oral medications in treating erectile dysfunction after a prostatectomy?
Are there any specific exercises or therapies that can help restore erectile function after a prostatectomy?
When should one consider surgical options like a penile prosthesis if other treatments are ineffective? Which factors should play a role in the decision making process?
2. Operative Technique of Penile Prosthesis Implantation
What does the surgery for implanting a penile prosthesis involve? Please describe the surgical steps.
What types of penile prostheses are available, and which might be best suited for me? What are the advantages and disadvantages for each type? Are there differences in the outcome?
How long is the recovery time after a penile prosthesis implantation? Are there differences for each type? When is the penile prosthesis ready for use after the operation?
What long-term risks are associated with penile prosthesis implantation and what are the most frequent complications?
How natural does an erection with a penile prosthesis feel?
3. Complications
What complications can arise after penile prosthesis implantation and what does the management of the complications involve?
How high is the risk of infection after penile prosthesis implantation and how is it treated?
Can penile prostheses shift or be damaged, and what action can be taken in such cases?
What is the risk of surgical revision after penile prosthesis implantation and how does revision affect the long-term outcome?
What are the signs of a complication after penile implantation? When should I go to the hospital?
4. Postoperative Care
What does typical postoperative care look like after penile prosthesis implantation?
What steps should I follow to ensure the best recovery after penile prosthesis implantation?
How often do penile prostheses need to be checked or replaced?
When can I return to normal work after the implantation of a penile prosthesis? What activities should I avoid?
Can I lead a normal sexual life after receiving a penile prosthesis, and are there any limitations or adjustments that I should be aware of? Please provide references.

**Table 2:** The evaluation of the reviewers’ answers to the questions with the DISCERN instrument. Each question was answered on a Likert scale from 0 to 5. Values are presented as mean ± standard deviation.

	ED and Treatment	PPI Surgery	Complications	Postoperative Care
Section A: Is the publication reliable?				
1. Are its aims clear?	4.53 ± 0.52	4.47 ± 0.52	4.34 ± 0.49	4.54 ± 0.52
2. Does it achieve its aims?	4.47 ± 0.52	4.4 ± 0.54	4.34 ± 0.49	4.47 ± 0.52
3. Is it relevant?	4.47 ± 0.52	4.43 ± 0.56	4.13 ± 0.35	4.27 ± 0.59
4. Is it clear what sources of information were used to compile the publication?	2.8 ± 1.37	1.87 ± 0.61	2.2 ± 1.08	1.94 ± 0.79
5. Is it clear on when the information used or reported in the publication was produced?	2.34 ± 0.98	1.73 ± 0.46	2.13 ± 0.83	2 ± 0.76
6. Is it balanced and unbiased?	3 ± 0.98	2.74 ± 0.88	2.74 ± 0.70	2.8 ± 0.56
7. Does it provide details of additional sources of support and information?	2.67 ± 1.38	2.13 ± 0.63	2.34 ± 0.72	2.4 ± 1.12
8. Does it refer to areas of uncertainty?	2.6 ± 0.82	2.24 ± 0.53	2.34 ± 0.82	2.6 ± 0.99
Section B: How good is the quality of information?				
9. Does it describe how each treatment works?	2.54 ± 0.75	2.3 ± 1.03	2.34 ± 0.89	2.2 ± 0.86
10. Does it describe the benefits of each treatment?	2.34 ± 0.82	2.4 ± 0.89	2.07 ± 0.88	2.2 ± 0.86
11. Does it describe the risks of each treatment?	1.8 ± 0.41	2.27 ± 0.68	2.67 ± 1.11	2.8 ± 1.08
12. Does it describe what would happen if no treatment is used?	1.67 ± 0.52	1.73 ± 0.59	1.87 ± 0.74	2.07 ± 0.88
13. Does it describe how the treatment choices affect overall quality of life?	2.14 ± 0.41	1.74 ± 0.49	1.87 ± 0.66	2.27 ± 0.80
14. Is it clear that there may be more than one possible treatment choice?	2.54 ± 1.22	1.9 ± 0.57	2.13 ± 0.99	2.07 ± 0.88
15. Does it provide support for shared decision-making?	1.97 ± 0.63	1.84 ± 0.69	1.94 ± 0.79	2.07 ± 0.88
Section C: Overall rating of the publication				
16. Based on the answers to all of the above questions, rate the overall quality of the publication as a source of information about treatment choices	2.74 ± 0.75	2.83 ± 0.75	2.94 ± 0.88	3.14 ± 0.64
Overall mean	2.79 ± 0.92	2.57 ± 0.98	2.65 ± 0.86	2.74 ± 0.90

Abbreviations: ED, erectile dysfunction; PPI, penile prosthesis implantation.

**Table 3:** Flesch Reading Ease scores (FRE) and Flesch–Kincaid Reading Grade Level scores (FKGL) according to subgroups of commonly asked questions prior to penile prosthesis implantation.

Section	FRE (0–100)	FKGL (US Grade Level)
ED and Treatment Options	9.8	17.41
Surgical Technique of Penile Prosthesis Implantation	17.80	16.18
Complications	19.02	15.99
Postoperative Care	28.39	14.04
Mean ± SD	18.75 ± 7.62	15.91 ± 1.40

Abbreviations: FRE, Flesch Reading Ease; FKGL, Flesch–Kincaid Reading Grade Level; US, United States; SD, standard deviation; ED, erectile dysfunction.

## Data Availability

The data associated with the paper are either available here, as stated in the section “Supplementary Materials”, or available from the corresponding author upon reasonable request.

## Supplementary Materials

The following supporting information can be downloaded at: https://www.mdpi.com/article/10.3390/jcm13247482/s1, Table S1. Comparison of ChatGPT’s answers to the EAU Guidelines on Sexual and Reproductive Health; Table S2. References provided by ChatGPT; Table S3: Raters’ Rationales for DISCERN Scores. Supplementary document: Questions and ChatGPT responses.
