# Supplementary material for: ChatGPT as a Support Tool for Informed Consent and Preoperative Patient Education Prior to Penile Prosthesis Implantation
Source: J Clin Med. 2024 Dec 10;13(24):7482. doi: 10.3390/jcm13247482 (PMC11679547; doi:10.3390/jcm13247482)
Supplement: Supplementary file 1 [file jcm-13-07482-s001.zip › jcm-3356373-supplementary.pdf]

Supplementary Table S1. Comparison of ChatGPT's answers to the EAU Guidelines on Sexual and Reproductive Health

Question	Answer of ChatGPT	Accuracy	Comparison to EAU Guidelines on Sexual and Reproductive Health 2024 [1]
**What are the most common causes of erectile dysfunction after a prostatectomy?**	Erectile dysfunction (ED) primarily results from nerve damage or blood vessel injury during the surgical removal of the prostate gland.	Yes	Matches EAU guideline
**What non-surgical treatment options are available for erectile dysfunction after a prostatectomy?**	Non-surgical options include oral medications (PDE5 inhibitors), vacuum erection devices, and penile injections.	Yes	Matches EAU guideline
**How effective are oral medications in treating erectile dysfunction after a prostatectomy?**	Effectiveness varies; more effective for nerve-sparing surgeries.	Yes	Matches EAU guideline, dependent on type of prostatectomy
**Are there any specific exercises or therapies that can help restore erectile function after a prostatectomy?**	Kegel exercises can help restore erectile function by strengthening the pelvic floor muscles.	Yes	Matches EAU guideline
**When should one consider surgical options like a penile prosthesis if other treatments are ineffective?**	Surgical options are considered after failure of non-surgical treatments.	Yes	Matches EAU guideline, dependent on individual factors
**What does the surgery for implanting a penile prosthesis involve?**	The surgery involves implanting either an inflatable or malleable penile prosthesis.	Yes	Matches EAU guideline
**What types of penile prostheses are available, and which might be best suited for me?**	Two main types: inflatable and malleable prostheses.	Yes	Matches EAU guideline, both types are recommended by EAU
**How long is the recovery time after a penile prosthesis implantation?**	Recovery generally takes 4-6 weeks.	Yes	Matches EAU guideline, timeframe is consistent
**What long term risks are associated with penile prosthesis implantation?**	Risks include infection, device malfunction, and penile shortening.	Yes	Matches EAU guideline, risks are described in EAU guidelines
**How natural does an erection with a penile prosthesis feel?**	Erections with inflatable prostheses are more natural compared to malleable ones.	Yes	Matches EAU guideline, dependent on type of prosthesis
**What complications can arise after penile prosthesis implantation?**	Complications include infection, mechanical failure, and erosion.	Yes	Matches EAU guideline
**How high is the risk of infection after penile prosthesis implantation?**	Risk of infection is around 0.3-2.7%, higher in diabetic patients.	Yes	Matches EAU guideline, dependent on health status
**Can penile prostheses shift or get damaged?**	Prostheses can shift or get damaged due to improper placement or physical trauma.	Yes	Matches EAU guideline, management is consistent with guidelines
**What does typical post-operative care look like after penile prosthesis implantation?**	Post-operative care includes avoiding strenuous activities and managing pain.	Yes	Matches EAU guideline
**How often do penile prostheses need to be checked or replaced?**	Check-ups are recommended at 3, 6, and 12 months, then annually.	Yes	Matches EAU guideline, regular

			check-ups are recommended
**When can I return to normal work after the implantation of a penile prosthesis?**	Normal work can be resumed within a few weeks.	Yes	Matches EAU guideline
**Can I lead a normal sexual life after getting a penile prosthesis?**	Sexual life can generally resume after recovery, but limitations may apply.	Yes	Matches EAU guideline, though individual adjustments may be necessary
**What steps should I follow to ensure the best recovery after penile prosthesis implantation?**	Follow surgeon's guidelines strictly to ensure the best recovery.	Yes	Matches EAU guideline
**What are the signs of a complication after penile implantation?**	Signs include pain, swelling, or malfunction of the prosthesis.	Yes	Matches EAU guideline
**What activities should I avoid after the implantation of a penile prosthesis?**	Avoid heavy lifting and strenuous activities for the first few weeks.	Yes	Matches EAU guideline, avoid strenuous activities

1. Salonia, A.; Bettocchi, C.; Capogrosso, P.; Carvalho, J.; Dinkelman-Smit, M.; Hatzichristodoulou, G.; Jones, T.H.; Kadioğlu, A.; Martinez-Salamanca, J.I.; Minhas, S.; et al. EAU GUIDELINES ON SEXUAL AND REPRODUCTIVE HEALTH.

Supplementary Table S2. References provided by ChatGPT

Question	Reference
**What are the most common causes of erectile dysfunction after a prostatectomy? Please provide References.**	https://www.hopkinsmedicine.org/health/conditions-and-diseases/prostate-cancer/erectile-dysfunction-after-prostate-cancer
	https://www.yalemedicine.org/clinical-keywords/erectile-dysfunction-after-radical-prostatectomy
**What non-surgical treatment options are available for erectile dysfunction after a prostatectomy? Please provide References.**	https://www.hopkinsmedicine.org/health/conditions-and-diseases/prostate-cancer/erectile-dysfunction-after-prostate-cancer
**How effective are oral medications in treating erectile dysfunction after a prostatectomy? Please provide References as a list.**	https://www.hopkinsmedicine.org/health/conditions-and-diseases/prostate-cancer/erectile-dysfunction-after-prostate-cancer
	Nature Reviews Urology: Weyne, E., et al., on "Landmarks in erectile function recovery after radical prostatectomy," reviews the impact of radical prostatectomy on erectile function and the role of oral medications in the recovery process.
	The Journal of Urology: Walsh, P.C., and Donker, P.J., provide insights into the etiology and prevention of impotence following radical prostatectomy
	European Urology: Montorsi, F., et al., discuss "Recovery of spontaneous erectile function after nerve-sparing radical retropubic prostatectomy with and without early intracavernous injections of alprostadil,"
**Are there any specific exercises or therapies that can help restore erectile function after a prostatectomy?**	https://health.clevelandclinic.org/post-prostate-surgery-kegel-exercises
**What types of penile prostheses are available, and which might be best suited for me? What are the advantages and disadvantages for each type? Are there differences in the outcome? Please provide a list of References.**	no exact references, only reference to journals (Journal of Sexual Medicine, Urology Journal, International Journal of Impotence)
**What long term risks are associated with penile prosthesis implantation and what are the most frequent complications? Please provide References.**	no exact references, only reference to journals and guidelines (EAU Guidelines, Journal of Sexual Medicine, Urology Journal, International Journal of Impotence)
**How high is the risk of infection after penile prosthesis implantation, and how is it treated? Please provide References.**	Gon, L.M., de Campos, C.C.C., Voris, B.R.I. *et al.* A systematic review of penile prosthesis infection and meta-analysis of diabetes mellitus role. *BMC Urol* 21, 35 (2021). https://doi.org/10.1186/s12894-020-00730-2
	Cosentino M, Bianco M, Ruiz-Castañé E, Iafrate M. Treatment of Penile Prosthesis Implant's Infection. Urol Int. 2020;104(7-8):542-545. doi: 10.1159/000508472. Epub 2020 Jun 15. PMID: 32541156.
**What is the risk of surgical revision after penile prosthesis implantation and how does revision affect the long-term outcome?**	Frydman, V., Pinar, U., Abdessater, M. *et al.* Long-term outcomes after penile prosthesis placement for the Management of Erectile Dysfunction: a single-Centre experience.*Basic Clin. Androl.* 31, 4 (2021). https://doi.org/10.1186/s12610-021-00123-x
**Can I lead a normal sexual life after getting a penile prosthesis, and are there any limitations or adjustments I should be aware of? Please provide References.**	https://www.smsna.org/patients/did-you-know/penile-implants-from-a-partner-s-perspective
	Chierigo F, Capogrosso P, Dehò F, Pozzi E, Schifano N, Belladelli F, Montorsi F, Salonia A. Long-Term

	Follow-Up After Penile Prosthesis Implantation-Survival and Quality of Life Outcomes. J Sex Med. 2019 Nov;16(11):1827-1833. doi: 10.1016/j.jsxm.2019.08.001. Epub 2019 Sep 6. PMID: 31501062.
	https://www.mayoclinic.org/tests-procedures/penile-implants/about/pac-20384916

Supplementary Table S3: Raters' Rationales for DISCERN Scores

DISCERN Criterion	Rater Rationale
3. Does it describe how each treatment works?	Responses were generally clear about mechanisms of action for non-surgical treatments, but surgical explanations lacked detail, especially regarding steps of the procedure.
4. Does it describe the benefits of each treatment?	Benefits were often highlighted effectively; however, responses sometimes lacked context, such as the expected timeframe for recovery or variability among patient outcomes.
5. Does it describe the risks of each treatment?	The model consistently mentioned common risks but failed to elaborate on rare or severe complications, leading to concerns about comprehensiveness.
6. Is it balanced and unbiased?	Responses tended to emphasize treatment benefits while underexplaining risks or alternatives. Some responses displayed a slight bias toward non-surgical treatments.
9. Does it provide support for shared decision-making?	Responses were inconsistent; while some answers encouraged consulting a healthcare provider, others lacked explicit support for shared decision-making processes.
12. Are the sources of information clear?	ChatGPT did not cite references, making it difficult to evaluate the reliability of the provided information. This lack of transparency lowered ratings for this criterion.
